# Supplementary material for: A Cancer-Specific Monoclonal Antibody against HER2 Exerts Antitumor Activities in Human Breast Cancer Xenograft Models
Source: Int J Mol Sci. 2024 Feb 5;25(3):1941. doi: 10.3390/ijms25031941 (PMC10856767; doi:10.3390/ijms25031941)
Supplement: Supplementary file 1 [file ijms-25-01941-s001.zip › ijms-2849198-supplementary.pdf]

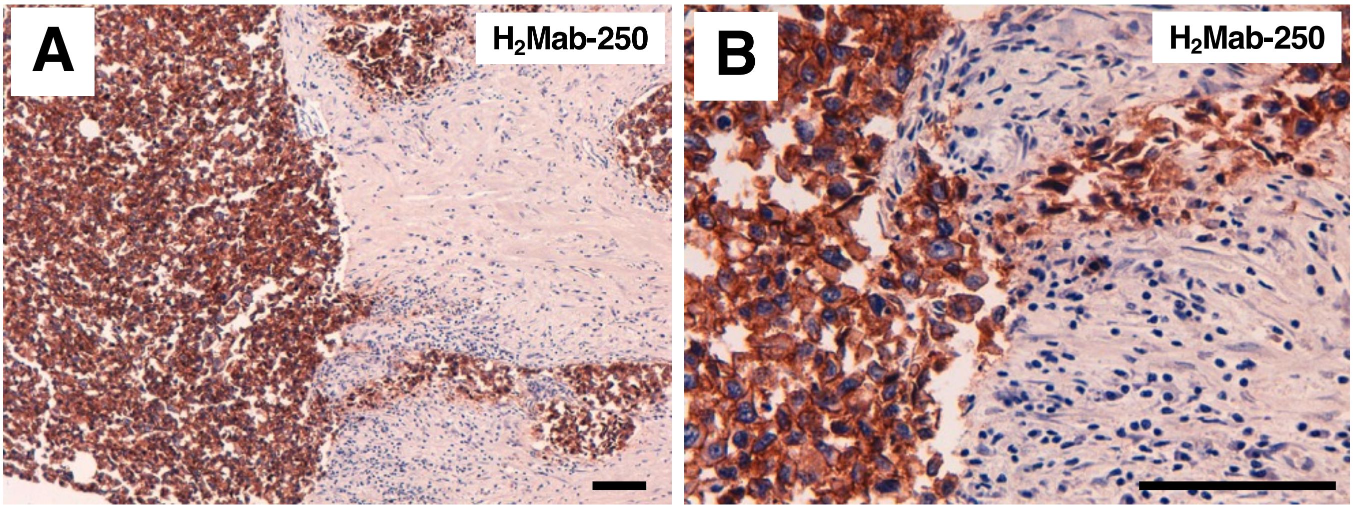

Supplemental Figure S1 Immunohistochemical analysis of H_2Mab-250 in a breast cancer tissue section without antigen retrieval. A HER2-positive breast cancer tissue sections were treated with H_2Mab-250 (1 μg/mL; **A, B**). The sections were then treated with the Envision+ kit. The chromogenic reaction was performed using DAB, and the sections were counterstained with hematoxylin. Scale bar = 100 μm.
